# Ethical Issues Regarding Dermatopathology Care for Service-Members: A Review

**DOI:** 10.3390/dermatopathology11040027

**Published:** 2024-09-24

**Authors:** Samir Kamat, Ross O’Hagan, Catherine Brahe, Curtis L. Hardy, Vikas Shrivastava, Jane M. Grant-Kels, Angela M. Crotty

**Affiliations:** 1 Department of Dermatology, Naval Medical Center San Diego, San Diego, CA 92134, USA; 2 Department of Dermatology, Icahn School of Medicine at Mount Sinai, New York, NY 10029, USA; 3 Department of Dermatology, University of Connecticut, Farmington, CT 06269, USA; grant@uchc.edu; 4 Department of Dermatology, University of Florida, Gainesville, FL 32611, USA; 5 Department of Dermatology, Naval Hospital Okinawa, Okinawa 901-2202, Japan

**Keywords:** dermatopathology, ethics, military dermatology, legal

## Abstract

Dermatologic care within the military faces unique ethical challenges. Service members are stationed across nationally and globally diverse settings, and therefore, dermatologic care rendered ranges from within resource-rich, advanced military medical treatment facilities to austere, resource-limited, deployed field environments. Additionally, military service members are often at unique risk for dermatologic disease, given occupational, environmental, and geographic exposures not commonly faced by their civilian counterparts. This review explores topics in dermatoethics via case analyses of ethical considerations within the scope of dermatologic care for military service members.

## 1. Background

Historically, ethical issues in dermatopathology have included approaches to client billing, appropriate qualifications for who should be interpreting dermatopathology specimens, and the inclusion of treatment suggestions in pathology reports, among others [1,2]. A recent 2022 survey of the American Society of Dermatopathology identified three ethical themes that dermatopathology must contend with: (1) financial influence (e.g., conflicts of interest, in-office dermatopathology laboratory), (2) work environment (e.g., conflict management), and (3) patient rights and provider obligations (e.g., transparency of cost, error reporting) [3,4]. Formal dermatoethics education has demonstrated promise in promoting ethical decisions. For example, a dermatoethics module at two academic institutions had a positive reception, with 91% recommending the modality to others and 31% “learning a great deal” [5]. Ethical considerations in dermatopathology may have more nuance in specific subpopulations, such as geriatric patients [6] or military service members, as is the focus of this review.

## 2. Objectives

- Provide an understanding of military dermatology and the unique constraints confronting a clinical provider.
- Equip readers and learners with an enhanced toolkit of ethical decision-making when caring for military service members.

## 3. Military Service Members: A Unique Patient Population

Military medicine focuses on patient care and mission readiness, which ensures that military forces can “fight tonight” [7]. However, military service can lead to flares of existing dermatologic diseases, the development of new conditions, or endemic infections [8].

Dermatologic disease exacerbation adversely affects both the patient and their broader military unit [9]. A patient who experiences treatment-refractory symptoms that disrupt their military jobs may be found unfit for service [10]. Examples of work impairment include an active duty military service patient with psoriasis who returns home early due to an in-field flare, a pilot with pruritis operating an aircraft, or an infantry soldier unable to wear their necessary protective headgear due to scalp psoriasis [11]. Treatment prospects may also be limited on deployment, given the lack of reliable lab monitoring and refrigeration availability [12]. The initiation of specific treatments, such as immunomodulators, may result in a medical evaluation for overall fitness for duty [13]. Regarding the broader unit, cutaneous exacerbations can involve medical evacuations, incur significant financial costs, and broadly affect mission readiness [9]. Adding further complexity, these members often have specialized roles that cannot be readily substituted [14].

Military occupations have increased risks of specific cutaneous injuries based solely on unique environmental conditions. For example, pilots and aerospace crews have increased susceptibility to solar cutaneous injury due to a lack of awareness of risk, low rates of sun protection, prolonged periods spent outdoors, and associated chronic sun exposure [15,16]. Conversely, service members working in cold-temperature climates can lead to associated cold-weather dermatologic injuries, including frostbite, immersion, pernio, Reynaud’s, and cold urticaria [17,18]. On the other end of the temperature spectrum, warm, humid climates can result in intractable and debilitating foot maceration and infections.

## 4. Ethics in the Dermatology Care of Service Members

Through case-based analysis [19], we will analyze six common dermatoethics scenarios encountered in the care of service members. The first three focus exclusively on dermatopathology; the last three are more broadly dermatology-related. These are also relevant because dermatopathologists may be in medical or surgical dermatology or leadership roles. Additional in-person discussions can enhance learners’ learning experience.

### 4.1. Clinical Ethics: Fundamental Values

Clinical ethics is often framed through the four key ethical principles—beneficence, non-maleficence, justice, and autonomy—from Beauchamp and Childress’s seminal work, “The Principles of Biomedical Ethics” [20]. (Summarized in Table 1). The principles have a prima facie obligation to be met when practicing medicine [21]. Prima facie obligations, conceptually originating from W.D. Ross’s ‘The Right and the Good’, are those for which there is some moral, self-evident basis [22]. These include, among others, fidelity, gratitude, reparations, and the four pertinent within this article—beneficence, non-maleficence, autonomy, and justice [22]. In actual situations, these prima facie duties frequently conflict; careful consideration, e.g., balancing and weighing these principles, must dictate the most prevailing duty and course of action. The following section summarizes the four fundamental ethical values that should account for all potential ethical issues involving patients, clinical providers (nurse, physician, advanced provider practitioner), and non-clinical staff (social workers, clinic staff).

i. Non-maleficence

Non-maleficence refers to not causing intentional harm; thus, the consequent behaviors are obligatory and colloquially understood by the “Do no harm” axiom in medicine [23]. This ethical value is codified in codes of conduct, the notion of negligence, and the risk aspect in the “risk–benefit ratio” [23]. Examples of non-maleficence in the workplace involve not exposing employees to harm without the appropriate protection and limiting dangerous working conditions, bullying, workplace violence, and otherwise poor work standards (poor wages, long hours, poor interpersonal relationships) [24].

ii. Beneficence

Beneficence refers to acting for an individual’s welfare [23]. The commitments arising from beneficence are not always salient and, therefore, not obviously obligatory, unlike those following the ethical value of non-maleficence [23].

iii. Autonomy

Autonomy refers to freedom from undue influence and the capacity to understand and act [23]. It is worth noting that autonomy is not always meaningfully relevant to patients who lack the capacity for such discernment, including infants, children, and those suffering from certain disorders [21]. In such cases, a surrogate decision-maker may make decisions on the patient’s behalf, using the substituted judgment standard (what the patient would have preferred in a given circumstance) or best interests standards (what is best for a patient, accounting for relative benefits and risks) [21]. Autonomy includes respecting the dignity of patients and providers, which is linked to informed consent, truth-telling, and privacy/confidentiality.

- Informed consent
  ○ Informed consent includes (1) competency, (2) full disclosure, (3) comprehension of disclosure, (4) acts of own volition, and (5) consent to the action in question [21].
- Truth-telling
  ○ Truth-telling refers to communicating with veracity and respecting a patient’s decision to know their health status (diagnosis/prognosis) or forgo this knowledge [21]. Truthfulness extends to interactions with family members and other providers and in charting.
- Confidentiality/Patient Privacy
  ○ Confidentiality refers to ensuring a patient’s health information is not disclosed outside the patient-physician relationship (except consultants) without appropriate patient consent, barring legally mandated disclosures or potential harm to others [21].

iv. Justice

Justice refers to the fair distribution of benefits and burdens, particularly relevant in resource-limited settings, global health, and healthcare access [23]. Difficulty in implementing justice often arises from identifying the most pertinent and fair distribution strategies. These strategies include considering equal share, need, effort, contribution, merit, and free-market outcomes [21]. Given the common involvement of resource-limited settings, this ethical value remains especially relevant to military medicine and clinical dermatology.

### 4.2. Existing Literature on Ethics and Military Dermatology

The following section summarizes the existing literature on military dermatology and clinical ethics.

Dermatologists, civilian and military alike, have special considerations for active duty service members, including the diagnostic and therapeutic implications for the member and their career, comprehensive documentation to inform medical readiness and potential benefits, and contributing to the robustness of a nation’s military force [10,25]. Militaries prioritize retaining a medically ready force; therefore, fitness for duty assessments is integral to medical providers’ responsibilities. Fitness for duty refers to whether a member’s physical disability appreciably impairs their ability to carry out military duties or whether medical accommodations are unreasonably prohibitive on the military [10]. In other words, diagnosis and treatment characteristics weigh on one’s medical readiness.

On the other hand, veteran care focuses on rendering care for the service-connected health needs of veterans after they have completed their military service. Most dermatologists may be familiar with caring for veterans in the Veterans Healthcare Administration (VHA), given the widespread prevalence of VA hospitals nationwide and the close connection to medical education [26]. Many have found purpose in caring for this population, perhaps prioritizing residency training, patient care (especially in private practice), and advocacy (increased VA funding) for veterans [26].

The healthcare systems serving active-duty service members and veterans are complex, integrated delivery systems with analogous challenges. In dermatology, variations in treatment availability (e.g., phototherapy, teledermatology, laser/photodynamic therapy, Mohs surgery) affect care delivery. Likewise, provider payment parity affects recruitment and retention [27]. When these respective systems cannot deliver care in a timely fashion, patients may receive indicated care in the community [27,28].

This range of activities begets ethical challenges, as previously discussed in the literature (Table 2). These prior analyses have included cases akin to this article that have focused on stereotyping, wait times, service-connected disabilities, requests for non-indicated care, subpar dermatology care, obligations to veterans, deployment, retirement, and limited follow-up, among others. Although varied, they all converge on the fundamental ethical principles that help chart the best course of action. The best solutions are often among the most moderate choices, e.g., conservative treatments, setting boundaries, understanding patient intentions and goals, etc.

### 4.3. Dermatopathology

The following three examples pertain specifically to dermatopathology, including (1) dermatopathology diagnosis, (2) the limited availability of dermatopathology slides, and (3) the role of specialized testing.

#### 4.3.1. Dermatopathology Diagnoses for AD Service Members

*I am reviewing a dermatopathology report that reads, “hyperkeratosis, regular acanthosis with neutrophils in the stratum corneum, and negative PAS/GMS stains, otherwise unspecified. Clinically correlate”. What are the ethical implications for under-calling psoriasis?*

Diagnosis in dermatology may not always be salient. For example, histopathological and immunofluorescence may reveal discordant findings or serve little utility in complex medical cases [30]. In addition, many histopathological features may be shared among benign and malignant lesions, such as melanocytic lesions [31]. In the case of psoriasis, while it can be diagnosed clinically, dermatopathology confirmation can be advantageous in this setting. Dermatopathology adds independent confirmation atop clinical judgment and may provide clarification under ambiguous circumstances. However, when clinical judgment and dermatopathology disagree, or the final diagnosis is unclear, there are ethical considerations on how to label a condition. This is particularly true in the military, where inflammatory conditions and therapeutic options impact active-duty status.

Accurate diagnosis reflects the ethical values of truthfulness and transparency that are fundamental to the operation of our healthcare system. The diagnosis on record is used by providers, payers, and patients for different but important purposes. In the military, diagnosis provides a sense of a member’s medical readiness, especially relevant if a member is ready to deploy soon. A general medical officer may adjust their pre-deployment planning, accounting for a patient with known psoriasis or potentially selecting alternate personnel if that disease is not well controlled. Likewise, an accurate diagnosis and documented severity can facilitate a medical waiver for a military recruit with a potentially disqualifying inflammatory skin condition [14].

The complete, transparent disclosure of a diagnosis also reinforces patient autonomy and enhances the choice of appropriate therapy. Misdiagnosis can compromise the therapeutic relationship and result in enhanced patient anxiety. Under calling psoriasis when there is doubt about the correct diagnosis may promote beneficence, specifically within the military setting, as it acknowledges diagnostic uncertainty and potentially avoids documentation of the wrong diagnosis. Accordingly, the dermatologist may pursue topical treatments in equivocal cases. This treatment course will likely have fewer implications regarding work or deployment-related restrictions, which upholds the patient’s dignity within the military and minimizes stigma for this member within their work environment.

Conversely, patients without a documented psoriasis diagnosis will likely be ineligible for specific systemic therapies with robust skin clearance rates of psoriatic lesions [32]. One treatment choice could be apremilast, a systemic small-molecule therapy. Although not the first line in the civilian sector, apremilast promotes beneficence as a systemic oral therapy within the military, minimizes non-maleficence with its limited side-effect profile, and upholds autonomy in preserving career opportunities [33].

In this scenario, the dermatologist is advised to consider carefully reconciling the clinical and dermatopathological findings. Full disclosure to the patient regarding the uncertainty and trialing certain medications for diagnostic and treatment purposes may promote beneficence in potentially mitigating some of the bothersome symptoms while also providing diagnostic clarity.

#### 4.3.2. Limited Availability of Dermatopathology Slides

*I am a Mohs surgeon and cannot access the patient’s pathology slides or clinical photos of the patient’s lesion that the referring provider biopsied. All I have received is the pathology report with the diagnosis of a cutaneous malignancy. How should I move forward?*

Military pilots as young as 20/30 have an increased risk for skin cancer due to chronic ultraviolet exposure [34]. This baseline vulnerability remains despite increased educational efforts regarding sunscreen use, protective clothing, etc. Mohs surgery has a very high cure rate, but a review of the tumor diagnosis and evaluation of its features on pathology by the surgeon is standard of care and helps the surgeon strategize their approach. Ethical ambiguity exists on the correct next step without the critical background slides.

In the military setting, taking a clinical photo as part of the medical documentation in this scenario is a Defense Health Agency (DHA) best practice; however, a clinical image taken downrange may be challenging to obtain and upload into the electronic health record (EHR). Additionally, a DOD- and HIPAA-compliant camera or smartphone capability may not be available to the downrange provider. Likewise, documentation, photos, and/or slides may not be readily accessible if patients receive care across different healthcare systems, and there are interoperability issues. Without these source materials, there is an increased reliance on pathology reports and patient-reported history.

Not having the opportunity to review the original slides and depending upon only the pathology report is possible but perhaps not in the patient’s best interest. If the initial sign-out were from a general pathologist, the report would likely not reflect the same nuance as one from a seasoned, board-certified dermatopathologist [35]. A retrospective study of pathology samples at one hospital found nearly a fifth to be solely descriptive [36]. In these circumstances, the clinical risk to patients may increase with the right confluence of factors, including underestimation, incomplete data, and absent clinicopathological correlation [36].

Another approach might be to re-biopsy the initial site to provide an initial understanding of the baseline residual pathology and morphology of the tumor. However, this may cause harm in exposing patients to a second biopsy procedure. Re-biopsying a lesion may also undermine justice, given the added time needed to take the biopsy, re-consider the approach, and overutilize limited resources. A second biopsy may even introduce anchoring bias if the dermatopathologists knew the first read [37]. These negative ethical consequences must be balanced with the potential beneficence of the second biopsy providing essential information.

Overall, one reasonable approach would be to refer the patient back or reach out via secure messaging to the initial referring provider asking them to provide more information and, in parallel, schedule a subsequent Mohs consultation at a future date. This strikes a balance between promoting beneficence by collecting additional key information and non-maleficence by not performing Mohs surgery in a potentially inappropriate clinical setting.

#### 4.3.3. Role of Specialized Testing

*An active-duty service member has skin lesions with pathology results that demonstrate a follicular neoplasm not otherwise unspecified. What are the ethical concerns for pursuing additional lab testing for a diagnosis that may otherwise be benign or represent an important syndrome?*

Dermatopathology may not offer clarity in some scenarios, and special stains (including immunoperoxidase stains) and other tests are needed to provide clarity for both patients and clinicians. This information is required to format the most appropriate treatment approach, whether proceeding toward additional testing, surgery, or escalation in medical therapy. Diagnostic clarity is given additional emphasis within the military because it may shape career placements. Thus, pursuing specialized lab testing for skin lesions must balance beneficence, autonomy, and justice.

In this scenario, the pathology report does not address whether the lesions are benign or malignant or could represent a cutaneous manifestation of a syndrome like Cowden or Birt–Hogg–Dubé. Thus, additional lab testing may uphold beneficence in providing more information and shaping optimal subsequent treatments. Special tests like genetic testing in the military can have positive effects, such as risk mitigation, ensuring fitness, and reducing downstream costs [38]. In dermatology, genetic testing may help provide further background on other susceptibilities for the patient and their relatives, such as familial atypical multiple melanoma syndrome [39].

Concurrently, patients retain autonomy to request more information and further workup of a medical condition and deserve full disclosure of their medical information. Medicine has moved away from the paternalistic approach, where the physician is the gatekeeper of information and tells the patient how they should be treated without consulting patients and their autonomous perspective. We now practice in a more patient-centric model of care, epitomized by the 21st Century Cures Act, which allows for visible, transparent notes available to patients. Within pathology reports, simplified language, explicit disclaimers for additional dermatologist counseling, and a lag between sign-out and patient release may help mitigate possible ensuing distress among patients who cannot comprehend the findings of specialized testing and become alarmed [40,41,42].

Autonomy and beneficence may reflect competing values in this scenario. Based on the idea that the benefit to the patient overrides their right to autonomy, the utilitarian school of thought may suggest pursuing specialized testing without explicit patient consent [43]. Conversely, a libertarian approach may suggest conducting informed consent (autonomy) before testing, even if it means a patient refusing additional testing and, therefore, missing a critical diagnosis [43].

Ordering specialized testing should also promote justice, particularly as these tests can be cost-prohibitive and may subtract from resources available to other patients. Military providers are afforded latitude to prioritize patients without some of the limitations seen in the civilian sector. For example, insulation from malpractice litigation, the lack of fee-for-service incentives, clinical decision support tools, and an integrated EMR system may limit the practice of defensive medicine often seen in civilian settings [44] The ability to practice without such restrictions or incentives implies a relative focus on the patient and pursuing what is important. However, predictive genetic testing that does not meet the standard of scientific criteria should be avoided even in the military [38].

We recommend pursuing specialized testing for patients with an ambiguous pathology report and clinically warrant it. In doing so, dermatologists should practice high-value care, understand the implications of their testing for management, and understand that patients may know the results of this testing before the provider.

### 4.4. Broader Dermatology

The following three examples in this second half pertain to more broad dermatology examples. Understanding the ethical challenges a dermatopathologist’s colleagues may encounter, or if they play multiple roles, will ensure a wider commitment to ethical practice. These include (4) laser therapy, (5) mental health and dermatology, and (6) collaterals as a military dermatologist.

#### 4.4.1. Laser Therapy for Wounded Warriors

*I do laser therapy for wounded warriors. What are the concerns about scar revision for more functional versus cosmetic goals?*

The military medical community’s application of lasers to treat patients with battle scars led to significant advances in laser surgery. While post-blast exposure is commonly associated with amputee care and prosthetic devices, combat veterans have also experienced considerable dermatologic burdens, both from functional deficits and cosmetic disfigurement due to injuries obtained on the front line. The original success of ablative fractional laser surgery in military dermatology treating battle scars led to widened use for scars from other types of injuries [45].

As with all limited resources, there are questions regarding which patients will benefit most and the appropriate time to initiate laser surgery. The decision to pursue laser therapy for medically indicated procedures reflects beneficence and standard of care. For example, laser therapy for laser hair removal for eligible and appropriate patients (such as those with pseudofolliculitis barbae) helps promote active duty standing and is a gold standard treatment [46,47]. For patients with battle wounds, laser therapy provides a salient visible benefit, affecting both psychological health as well as wound healing. Finally, laser therapy for conditions with increasing evidentiary base is indicated, such as for laser hair removal to reduce the recurrence of pilonidal cysts [48].

On the other hand, laser therapy in the military setting can also potentially compromise justice, as it is expensive and can strain resources. This is particularly true when used for cosmetic purposes. The military dermatologist, however, should have the autonomy to recommend and provide this kind of care when they believe it is appropriate. They may also benefit from maintaining their skill and remaining proficient long after residency training. In resource-constrained settings such as the military, accommodating an overwhelming demand for medical dermatology services versus provider autonomy and skill retention is an ever-present challenge. Further challenging is the grey zone of employing traditionally cosmetic modalities for medical-grade conditions, which may have varied insurance coverage, such as dermabrasion for acne [49].

Given the increasing role of media influencers and misinformation, there is increased demand for cosmetic services even in the military, resulting in requested care for non-medical indications. Accordingly, the ethics of cosmetic dermatology remain relevant, including patient autonomy (decision to influence self-image and subsequent confidence, informed consent, and expectation management) [50]. Military dermatologists must consider whether there is an actual medical necessity before delivering care. Further, despite the common misconception that the military health system will cover all medical services, service men and women may face personal financial charges for what might be identified as superfluous medical care. Educating military personnel and providing transparency on what will be covered versus what is considered cosmetic is required. Providers should also consider a member’s deployment schedules to prevent non-maleficence via mitigating complications, such as a member with facial hyperpigmentation possibly experiencing disease exacerbation in a sunny environment [29].

Therefore, when caring for service members who request or need laser therapy, military dermatologists should consider the medical indication, the availability of resources, and potential military-unique deployment schedules that may complicate the use of such devices.

#### 4.4.2. Mental Health and Dermatology

*An active-duty service member presents with severe inflammatory skin disease. What are the ethical commitments to screen for concurrent mental health disorders, particularly in a busy outpatient clinic?*

Dermatologic diseases have been associated with depression, anxiety, reduced self-esteem, and even delinquency [51,52]. Service members routinely encounter stressors that may exacerbate dermatologic disease and worsen mental health. These include high-tempo deployment schedules, constant relocating, being isolated from family/friends, and having to perform challenging jobs. The severity of the military health burden, reflected in the staggering 28.1% of all deaths in the US military due to suicide in 2013, is a serious operational and national security issue [53]. The rate of depression and suicide in our military suggests that we are all stakeholders in the vested challenge to confront this mental health epidemic, including dermatologists. Yet, given the general culture of self-sufficiency and self-reliance, missing psychological manifestations of cutaneous disease may be common [53].

The relationship between dermatologic disease and mental health takes on special significance within the military. Rosacea, acne, and hidradenitis suppurative (HS), all common in the military, have been linked to mental well-being [53]. Chronic sleep deprivation, like that seen in deployed settings, may lead to adverse immune regulation and poor psychological health, exacerbating dermatologic disease [54]. Atopic disease flares may be challenging to control in the setting of constant exposure to possible contact allergens (standard uniform and laundering, military gear), chronic stress, and short medication supplies [14]. HS management is complex in austere living conditions (ships, fields), where adequate hygiene, running water, or shower availability is inconsistently available [55]. Regular grooming upkeep may exacerbate hair disorders, such as acne keloidalis nuchae, pseudofollicultis barbae in men and, traction alopecia, tricorrhexis nodosa, and extracranial headaches in women [56,57]. For all these conditions, patients can be given limited duty assessments or treatments that can take them away from their core work priority (e.g., a pilot on minocycline or an AD patient on limited duty) [11]. Patients may, therefore, be reluctant to seek care or downplay their symptoms for professional reasons, thereby not receiving indicated treatment and concurrently experiencing prolonged stress.

The decision for a dermatologist to screen for mental health impairments is a multi-faceted one. Military personnel are vulnerable to mental health issues at baseline, and their dermatologist may bear the closest semblance of a primary care provider they are seeing regularly. Understanding the extent of the mental health burden can reinforce the therapeutic relationship and may inform subsequent referral to a mental health provider or escalation in medical treatment.

On the other hand, screening for concurrent mental health disease, while potentially beneficial, may broach the issue of the availability of subsequent appointments. The availability of subsequent counseling is often unclear and may reflect mission creep whereby dermatologists take on roles more within the scope of a primary care provider. Even if a patient discloses some mental health concerns, barring severe mental health requiring awareness of their commander or escalation, that patient may decide they do not want further support [11]. This may often be the case with service members who tend to self-manage their stressors, reflecting the ethical value of autonomy. Military personnel may also be concerned about having a mental health issue in their medical record, as it could impact deployment or promotion opportunities.

A dermatologist must use discretion and clinical judgment to decide whether to pursue further screening for mental health issues among their dermatologic patients. Overall, the dermatologist is advised to perform a simple PHQ-2 or employ a validated instrument, document that concern for subsequent visit counseling, and consider referral to a mental health provider. Fortunately, a well-done history of dermatologic and relevant mental health impacts can promote beneficence and mission readiness.

#### 4.4.3. Collaterals as a Military Dermatologist

*What are the ethical considerations for taking on collateral duties as a military dermatologist or dermatopathologist? I want to pursue other medical/leadership roles, but as a solo dermatologist/dermatopathologist for a large referral base, this may limit my access to patient care. What are the ethical commitments of a military dermatologist/dermatopathologist to participate in extra-clinical obligations, such as quality improvement projects, resident education, or leadership posts in their local command?*

With service members deployed across the globe, military dermatologists may fill a critical need for specialty care, particularly considering the prevalence of dermatologic complaints. However, military dermatologists also hold a dual role as military commissioned officers. They are expected to demonstrate all the customs and courtesies of all their military counterparts and contribute to their local command. Unlike their civilian counterparts, military dermatologists are routinely placed in positions that extend beyond clinical dermatology and emphasize skillsets often developed in their early medical training. For example, in austere environments or combat settings, dermatologists may play more roles as primary care physicians responsible for up to 300–1000 service members or even trauma-related care and have undergone certification in advanced trauma life support (e.g., tactical combat casualty care) [58].

Balancing these multiple roles can be challenging, and additional optional responsibilities can present an ethical challenge.

Clinical practice factors are increasingly impacting dermatologists, including the number of patients they see daily, the frequency of procedures, and the supervision of mid-level providers and other support staff. Nonetheless, dermatologists still retain dignity as providers and, therefore, the autonomy to set boundaries between their professional clinical responsibilities and whether to take on positions of increasing responsibility. For example, dermatologists may accept a leadership role that diminishes their clinical responsibility and substitute it with time devoted to other roles, such as serving on an ethics committee or leading a department or hospital.

A dermatologist participating in quality improvement projects can improve workflow and processes. Being involved with command leadership allows dermatologists to continue to grow professionally while advocating for their patients’ needs, such as the need for increasing resources, staff members, or supplies. This autonomy can improve justice by improving both the delivery and extent of patient care beyond their immediate clinical practice and, therefore, should be encouraged.

However, time spent on these other duties will affect patient access and justice, especially if no other dermatologist is available in the immediate area. Care must be prioritized to avoid non-maleficence, and commitments must be considered to existing patients. Fortunately, the advent of teledermatology has increased the scope of access and expanded the practical catchment areas of dermatology to include otherwise remote, austere regions. One teledermatology study between 2004 and 2012 found that teledermatology consultations were addressed in a timely 24-h period, and 23% were addressed within the first hour [59]. Another study identified the synchronous utility of teledermatology and teledermoscopy, which may further overcome remote challenges in patient access [60].

The best resolution strikes an ideal balance between upholding autonomy and justice with beneficence and non-maleficence. For optional, non-mandated commitments, military dermatologists are advised to carefully assess their time commitments and ensure they can care for their patients before assuming additional responsibilities.

## 5. Conclusions

The unique facets of military service lead to a unique risk profile for dermatologic disease and ethical challenges for the service members and their dermatologists. The clinical decision requires considering the availability of resources and overall mission readiness. Herein, we have reviewed military dermatoethics, specifically across six examples: (1) dermatopathology diagnoses, (2) limited dermatopathology resources, (3) the role of specialized dermatopathology testing, (4) laser dermatology, (5) mental health and dermatologic disease, and (6) collaterals as a military dermatologist/dermatopathologist. We hope that applying a case-based style will augment military dermatologists’ ethical toolkit for managing complex ethical situations.

## Figures and Tables

**Table 1:** Summary of ethical values.

Medical Ethical Values	Definition
Beneficence	To do good; patient’s well-being comes first
Non-maleficence	To do no harm, either unintentional or intentional
Justice	Equity in the delivery of care
Autonomy	Respecting the dignity of patients and providers via patient privacy, confidentiality, and truthfulness
Dignity	For the patient (and provider)
Truthfulness	Informed consent and truthfulness with family members, other providers, and in charting

**Table 2:** Literature Review of Dermato-ethics about military service-members and veterans.

Article Title	Year	Authors	Ethical Scenario	Reasonable Choice	Ethical Values
Ethical issues regarding caring for dermatology patients in the U.S. Department of Veterans [27] Affairs Health Care System	2012	Reich et al.	Patient upset about wait times insisting for an immediate appointment, possibly escalating to violence [27].	Establish boundaries and schedule at next available appointment.	Not reported
			Stereotyping veterans from different wars [27].	Develop a curious and open attitude.	Not reported
			Asserting dermatologic disease as service-connected [27].	Individual patient assessment, communication with patient, and comprehensive documentation.	Not reported
			Subpar performance of a treating provider [27].	Support for struggling dermatologist.	Not reported
			Request for non-indicated cosmetic care [27].	Individual assessment: perform intervention in accordance with Code of Federal Regulations, refer to cosmetic dermatologist, refer to mental health provider.	Not reported
Dermatologists ethical obligation to veterans [26]	2024	Donohue et al.	Not reported	Not reported	Not reported
Ethical concerns in caring for active duty service members who may be seeking dermatologic care outside the military soon [25]	2020	Dodd et al.	AD service member presents with basal cell nevus syndrome refuses treatment following prior treatment-associated cosmetic disfigurement and requests medical clearance for promotion [25].	Discuss treatment options and select most optimal choice for individual patient.	Paternalism, informed consent, patient autonomy
Dermatologic barriers to deployment: Ethical considerations when treating military service members [28]	2024	Donohue et al.	Systemic therapy for psoriasis potentially limiting deployability [28].	Devise treatment plan incorporating treatment options and effects on military service.	Beneficence, nonmaleficence
Considerations for dermatologists when treating U.S. Military Service Members	2017	Kels et al.	Service member approaching retirement and pension with unclear optimal treatment plan [10].	Err on side of starting more conservative therapy.	Beneficence, nonmaleficence
An Ethical Analysis of Treatment of an Active-Duty Service Member With Limited Follow-up	2024	Kamat et al.	Service member requesting care for skin dyspigmentation set to deploy soon [29].	Topical therapy with telemedicine follow-up.	Beneficence, justice, autonomy, non -maleficence
